# How do moth and butterfly taste?—Molecular basis of gustatory receptors in Lepidoptera

**DOI:** 10.1111/1744-7917.12718

**Published:** 2019-09-12

**Authors:** Wei Xu

**Affiliations:** ^1^ Agricultural Sciences College of Science, Health, Engineering and Education, Murdoch University WA Australia

**Keywords:** bitter receptor, CO_2_ receptor, GR43a, sugar receptor, taste receptor

## Abstract

Insect gustatory system plays a central role in guiding insect feeding behaviors, insect–plant interactions and coevolutions. Gustatory receptors (GRs) form the interface between the insect taste system and their environment. Previously, most studies on insect GRs are focused on *Drosophila*; much less attention has been paid to Lepidoptera species, which consist of a large number of serious agricultural crop pests. With the exceptional advances in the next generation sequencing (NGS), cellular biology, RNA interference (RNAi), and clustered regularly interspaced short palindromic repeats (CRISPR) technologies in recent years, extraordinary progresses have been achieved elucidating the molecular mechanisms of Lepidopteran GRs. In this review, we highlighted these advances, discussed what these advances have revealed and provide our new insights into this field.

## Introduction

**Fig. 1:**
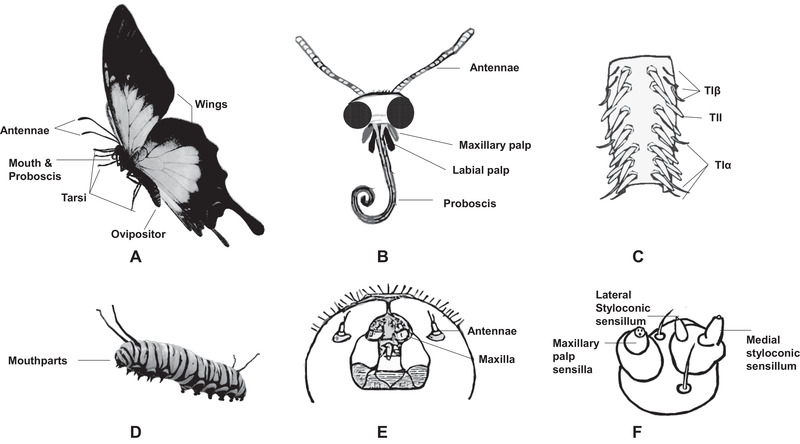
Major Lepidoptera gustatory tissues and gustatory sensilla on (A) adults and (B) larvae. Gustatory tissues on the adult heads (C) and larval heads (D). (E) Schematic diagram illustrating three different sensillum types of *S. littoralis* female adult tarsi: TIα, TIβ and TII (Seada *et al*., 2018). (F) Schematic diagram illustrating the different sensillum types of *B. mori* larval maxilla including maxillary palp sensilla, medial styloconic sensillum and lateral styloconic sensillum (Zhang *et al*., 2013).

Chemical communication is essential in guiding insect behaviors such as mating, foraging, host‐feeding, and oviposition. Chemosensory receptors act as an interface between insects and their chemical environment, which include transient receptor potential (TRP) channels, pickpocket (Ppk) channels, olfactory receptors (ORs), ionotropic receptors (IRs), and gustatory receptors (GRs). TRP are a group of ion channels that play a pivotal signaling role in virtually all sensory modalities. Recently, a TRP channel was discovered the target of certain feeding inhibitors, so they are considered as potential insecticide targets (Salgado, 2017). PpK, a subfamily of degenerin–epithelial sodium channels, are required for pheromone‐guided sexual behaviors in *Drosophila* (Joseph & Carlson, 2015). ORs are localized on the dendritic membrane of the olfactory sensilla, detect volatile compounds, and transduce the olfactory signals to insect brains to regulate behaviors (Fleischer *et al*., 2018). They function together with a highly conserved receptor called the odorant receptor coreceptor (Orco) (Sato *et al*., 2008; Wicher *et al*., 2008; Luo & Carlson, 2018). IRs are a variant subfamily of ionotropic glutamate receptors and function as ligand‐gated ion channels involved in chemosensation (Liu *et al*., 2018; Rimal & Lee, 2018). Previous studies on *Drosophila* IRs grouped them to two subfamilies, antennal IRs that are expressed mainly in the antennae and exclusively in neurons in coeloconic sensilla and divergent IRs that are generally expressed elsewhere (Rytz *et al*., 2013; Rimal & Lee, 2018). GRs are receptors housed inside gustatory sensilla, which are widely distributed on antennae, tarsi, mouthparts, wings, and ovipositors (Fig. 1). GRs can detect nonvolatile compounds including sugars, bitters, amino acids and plant secondary metabolites via contact chemosensation (Agnihotri *et al*., 2016). The ORs and GRs share a common amino acid residue motif at the C‐terminal domain, suggesting they have evolved from the same ancestral chemoreceptor genes (Robertson, 2019). Following studies further revealed that *Or* genes evolved from the *Gr* gene family (Robertson *et al*., 2003). Insect ORs and GRs were originally considered as a large group of G protein‐coupled receptors (GPCRs); however, subsequent *in vitro* studies have predicted that they have an inverted GPCR transmembrane structure (Benton *et al*., 2006; Zhang *et al*., 2011).

Insect *Gr* genes were first identified from the *Drosophila melanogaster* genome (Clyne *et al*., 2000), which have been classified into four clades based on the phylogeny and the ligands they detect. They are carbon dioxide (CO_2_) (Robertson & Kent, 2009), GR43a‐like (Sato *et al*., 2011), sugar (Slone *et al*., 2007; Kent & Robertson, 2009), and bitter (Wanner & Robertson, 2008) GR subfamilies. To date, GR research has been focused on *Drosophila* or mosquitoes, but with the advanced next generation sequencing (NGS) technologies and increasing availability of genomic information from other insect species, it is being extended to a diverse range of species (Kang *et al*., 2018; Robertson *et al*., 2018; Robertson *et al*., 2019). Especially the completed genome projects on Lepidopterans including *Bombyx mori* (Wanner & Robertson, 2008), *Danaus plexippus* (Zhan *et al*., 2011), *Heliconius melpomene* (Briscoe *et al*., 2013), *Plutella xylostella* (You *et al*., 2013), *Manduca sexta* (Kanost *et al*., 2016), *Helicoverpa armigera* (Pearce *et al*., 2017), and *Spodoptera frugiperda* (Gouin *et al*., 2017) provide us invaluable resources to gain an insight into the Lepidopteran GRs (Agnihotri *et al*., 2016; Robertson, 2019).

## The number of GRs

Various insect species consist of different numbers of GRs. The expansion of *Gr* genes is mainly caused by a high number of gene gains and relatively few gene losses (Engsontia *et al*., 2014). The total number of GRs may be linked to an insect species’ behavior and ecology. Studies suggest *Gr* and *Or* genes are close in the numbers in many insect species’ genomes. For example, *D. melanogaster* has 60 *Or* and 60 *Gr* genes. *Anopheles gambiae* has 79 *Or* and 76 *Gr* genes. *Acythosiphon pisum* has 79 *Or* and 77 *Gr* genes. *Tribolium castaneum* has 261 *Or* and 215 *Gr* genes. Human louse (*Pediculus humanus*) has only six *Gr* genes, such a low number may be related to its obligate ectoparasite of humans (Table 1) (Xu *et al*., 2016; Guo *et al*., 2017). An exception is the Hymenoptera families including ants, bees and wasps, which have much more *Or* genes than *Gr* genes. Honeybee, *Apis mellifera*, has 163 *Or* genes but only 12 *Gr* genes (Robertson & Wanner, 2006), presumably due to its typical foraging and social behaviors (Table 1) (Robertson & Wanner, 2006). Another exception is *H. armigera*, which has an expansion of 197 *Gr* genes (Xu *et al*., 2016). Comparing to other specialist Lepidoptera species, 76 GRs were identified from *B. mori* (Wanner & Robertson, 2008), 58 GRs were identified from *D. plexippu*s (Zhan *et al*., 2011), 72 GRs were identified from *H. melpomene* (Dasmahapatra *et al*., 2012), and 69 GRs were identified from *P. xylostella* (Table 1) (You *et al*., 2013). These four species are all specialist feeders: *B. mori* is a mulberry leave specialist (Wanner & Robertson, 2008); *D. plexippus* consumes only plants in the milkweed family (Asclepiadacea) (Zhan *et al*., 2011); *H. melpomene* feeds on either *Passiflora oerstedii* or *Passiflora menispermifolia* (Dasmahapatra *et al*., 2012); and *P. xylostella* feeds exclusively on crucifers (You *et al*., 2013). Therefore, the expansion of 197 GRs may be linked to *H. armigera* capacity for being a successful generalist, which presumably broadens the sensation range of plant secondary metabolites (Xu *et al*., 2016). The comparative study on GRs between specialist and generalist Lepidopterans will shed light on the Lepidoptera–plant interactions.

**Table 1:** The Numbers of olfactory receptors (ORs) and gustatory receptors (GRs) genes identified from genome sequences of selected insect species

Insect species	ORs	GRs
*Helicoverpa armigera*	64	197
*Plutella xylostella*	95	69
*Bombyx mori*	68	76
*Heliconius melpomene*	69	72
*Danaus plexippus*	64	58
*Aedes aegypti*	113	95
*Anopheles gambiae*	79	76
*Drosophila melanogaster*	60	60
*Tribolium castaneum*	261	215
*Pogonomyrmex barbatus*	344	73
*Apis mellifera*	163	12
*Nasonia vitripennis*	301	58
*Acyrthosiphon pisum*	79	77
*Pediculus humanus*	10	6
*Locusta migratoria*	95	75

## The distribution of GRs

GRs are expressed inside gustatory sensilla on the gustatory tissues including antennae, proboscises, maxillary palps, labial palps, tarsi, wings, and ovipositors (Figs. 1A–D). Structurally different sensilla have been discovered from these gustatory tissues, which often play different roles in sensations. For example, the proboscis sensilla in Lepidoptera can be divided into six types according to their external morphology: sensilla chaetica, sensilla basiconica, sensilla styloconica, sensilla coeloconica, sensilla filiformia, and sensilla campaniformia (Krenn, 2010). Morphological and functional characterization of insect gustatory sensilla could lead to an improved understanding of the mechanisms underlying the acceptance and rejection of resources, and the gustatory receptor neurons (GRNs) inside the sensilla. In *Spodoptera littoralis* female adult tarsi, by using morphological and electrophysiological analyses on each sensillum, three distinct functional classes (TIα, TIβ, TII) were characterized based on the response spectra of three of the four responding GRNs (Fig. 1E) (Seada *et al*., 2018). A comparative study between *B. mori* and *H. armigera* larvae showed no obvious morphological differences of either the styloconic sensilla on the maxillary galea or the basiconic sensilla on the maxillary palp (Fig. 1F) (Zhang *et al*., 2013). However, *myo*‐inositol and sucrose were detected specifically by two GRNs located in *B. mori* lateral styloconic sensillum, whereas in *H. armigera*, sucrose was sensed by a GRN in the lateral sensillum, and *myo*‐inositol by a GRN in the medial sensillum (Zhang *et al*., 2013).

The advanced RNAseq analysis of diverse chemosensory organs of larvae and adults yielded precise maps of GR expression in various organs of Lepidoptera species such as *B. mori*, *H. armigera*, and *H. melpomene* (Briscoe *et al*., 2013; Xu *et al*., 2016; Guo *et al*., 2017). For example, most *H. armigera Gr* genes detected at adult stage were found in heads, abdomens or female ovaries. Certain individual *Gr* genes were expressed in multiple tissues, but some *Gr* genes showed expression limited to specific developmental stages or organs and tissues. For example, HarmGR35 was only detected in adult heads while HarmGR65 was detected in larval fat bodies and male adult abdomens. HarmGR195 was only detected in adult tarsi (Xu *et al*., 2016). In *H. melpomene*, 26 GRs showed female‐biased gene expression and 21 of them are *Heliconius*‐specific, which is coincided with an obvious sexual dimorphism in the abundance of gustatory sensilla on the *H. melpomene* forelegs, suggesting that female oviposition behavior may drive the evolution of new GRs in butterfly genome (Briscoe *et al*., 2013). The expression profile of *B. mori* GRs was analyzed by using RNA‐seq in various chemosensory organs of larvae and adults (Guo *et al*., 2017). Interestingly, many GRs, especially bitter GRs were found clustered in the same chromosomes such as Chromosome 7 and 13, which were detected expression in the same tissues. Forty‐six *BmorGr* genes were expressed in larval maxillae, 44 *BmorGr* genes were expressed in larval thoracic legs and 52 *BmorGr* genes were expressed in adult legs, indicating that these appendages are important gustatory tissues. BmorGR63 showed high expression levels in all organs at both larval and adult stages. By contrast, BmorGR19 was highly expressed in larval chemosensory organs (especially antennae and thoracic legs). BmorGR53 and BmorGR67 were expressed exclusively in larval tissues. *BmorGr27*−*BmorGr31* genes were clustered on chromosome 7 and showed a high expression level limited to adult legs (Guo *et al*., 2017).

## Functional category of GRs

Based on phylogeny and the types of the ligands they can detect, insect GRs were classified into CO_2_, sugar, GR43a‐like, and bitter receptor subfamilies.

### CO_2_ receptors

CO_2_ is ubiquitous in the environment and plays critical roles in insect life. Insects require sensitive and robust systems to detect environmental CO_2_, which is responsible for regulating diverse behaviors in different insect species. For example, in the densely populated nests of social insects like bees, wasps, ants, and termites, the CO_2_ concentration is much higher than the atmospheric concentration. These social insects may use CO_2_ to help locate their nests (Seeley, 1974). For mosquitoes, CO_2_ is a cue for locating hosts for blood feeding, so it is often utilized as an effective attractant in mosquito traps (Syed & Leal, 2007; Guerenstein & Hildebrand, 2008). For moths, CO_2_ gradients may indicate floral quality. Fresh flowers, which may provide better nectar release, produce more CO_2_ than older flowers (Thom *et al*., 2004).

Insect CO_2_ receptors were first identified from *Drosophila* antennae. DmelGR21a and DmelGR63a have been shown to be required for CO_2_ detection (Suh *et al*., 2004; Jones *et al*., 2007; Kwon *et al*., 2007). CO_2_ GRs are conserved across many insect species (Wanner & Robertson, 2008; Erdelyan *et al*., 2012; Briscoe *et al*., 2013). However, no genes orthologous to the CO_2_ GRs have been identified from the genomes of honeybee, wasp, ant, pea aphid, blacklegged ticks (*Ixodes scapularis*), human louse, or locust (Robertson & Kent, 2009; Wang *et al*., 2014). Honeybees and blacklegged ticks are known to respond to CO_2_ physiologically, indicating they may have another pathway for CO_2_ detection (Robertson & Kent, 2009; Wang *et al*., 2014).

**Fig. 2:**
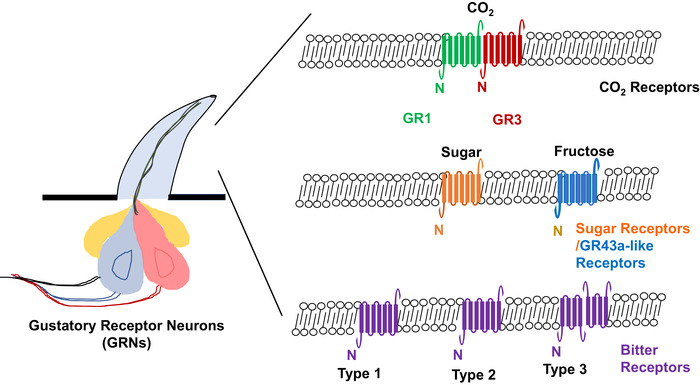
Molecular mechanism of Lepidopteran gustatory receptors (GRs) including CO_2_, sugar, GR43a‐like, and bitter GRs in gustatory sensilla as discussed in this review. (A) CO_2_ receptors. Two Lepidopteran GRs, GR1 and GR3, are indispensable and sufficient for CO_2_ sensing. (B) Sugar receptors and GR43a‐like receptors. Both two types of GRs show an inverted topology relative to GPCR with an intracellular N‐terminus and an extracellular C‐terminus. They can detect sugars such as *myo*‐inositol and D‐fructose. (C) Bitter receptors. Three types of bitter GRs have been identified. Members of type1 and type 2 GRs have been shown responses to feeding deterrents such as coumarin and caffeine. Type 3 GRs showed two new topologies: both C and N‐terminus intracellular or extracellular. One Type 3 GR (HarmGR195) showed response to proline.

Among the insect species that have CO_2_ GRs, the numbers of genes present in the genomes are variable (Kwon *et al*., 2007; Robertson & Kent, 2009). Unlike *Drosophila* has two CO_2_ GRs, beetles, moths, butterflies and mosquitoes possess three (Robertson & Kent, 2009; Briscoe *et al*., 2013). CO_2_ receptors have been functionally characterized *in vivo* using transgenic *Drosophila*, mutated mosquitoes or RNAi techniques (Jones *et al*., 2007; Kwon *et al*., 2007; Cayirlioglu *et al*., 2008; Hartl *et al*., 2011; Erdelyan *et al*., 2012; McMeniman *et al*., 2014). Three GRs (HarmGR1, HarmGR2, and HarmGR3) were identified as orthologues of CO_2_ receptors from *H. armigera* labial palps; the organs detect CO_2_ in moths (Xu & Anderson, 2015; Ning *et al*., 2016). By using *in situ* hybridization, the expression of HarmGR1, HarmGR2, and HarmGR3 were further detected coexpressed in the same cells of the labial palps (Ning *et al*., 2016; Guo *et al*., 2018). HarmGR2 was also detected in the adult thoraxes, female adult tarsi, male adult abdomens, adult antennae, larvae hindgut and larvae ventral nerve, suggesting it may have other functions (Xu & Anderson, 2015). By using Sf9 cells coupled with calcium imaging system, HarmGR3 showed a dose‐dependent response to sodium bicarbonate (NaHCO_3_) individually while the other two, HarmGR1 and HarmGR2, did not (Xu & Anderson, 2015). However, by using the *Xenopus* oocytes expression and the single cell recording system, only oocytes coexpressing HarmGR1/HarmGR3 or HarmGR1/HarmGR2/HarmGR3 gave robust responses to NaHCO_3_, uncovers that HarmGR1 and 3 are indispensable and sufficient for CO_2_ sensing (Fig. 2) (Ning *et al*., 2016). These different results may be caused by two different systems. Sf9 cells are derived from ovarianies (*Spodoptera frugiperda*), so they may possess a native receptor that can couple with HarmGR3 in detection of NaHCO_3_.

### Sugar receptors

Sugars play critical roles in insect life as valuable energy and food resources. The perception of sugars is always utilized by insects to assess and evaluate the nutritional value of foods (Slone *et al*., 2007; Kent & Robertson, 2009). For example, bees collect nectar from blossoms in the field and convert them into honey, which contains a high level of sugars (Pham‐Delegue *et al*., 1990); mosquitoes utilize sugars from flowers and plants as an energy source (Foster, 1995); aphids produce sugar‐rich honeydew for ants to eat and ants protect aphids from natural enemies, such as lady bugs and wasps (Perkins *et al*., 2013); populations of the German cockroach have evolved an adaptive behavioral aversion to glucose, a phagostimulant component of toxic baits (Wada‐Katsumata *et al*., 2013). Therefore, insects require sensitive, accurate, and robust sensory systems to detect sugars and regulate these diverse behaviors across different insect species (Slone *et al*., 2007; Kent & Robertson, 2009). Interestingly, some insect species lost their sugar receptor family in the genome, for example, *Glossina morsitans*, *P. humanus*, *Rodnius prolixus*, and the bedbug *Cimex lectularium*. A possible reason is that all of them are obligate blood feeders. Similar phenomenon was observed from the domestic cat, which showed no response to sucrose and several other sugars (Li *et al*., 2005).

The “GR43a‐like” receptors have been shown to actively respond to fructose or inositol, but they will be discussed separately in this review (Sato *et al*., 2011; Xu *et al*., 2012). Eight *Gr* genes have been mapped to *Drosophila* sweet taste neurons, defining a distinct clade of sugar receptors (Dahanukar *et al*., 2007; Jiao *et al*., 2007; Slone *et al*., 2007; Jiao *et al*., 2008), whose orthologous genes are found across various insect species (Kent & Robertson, 2009). Five sugar GRs (BmorGR4–8) were identified from *B. mori* (Wanner & Robertson, 2008). By using RT‐PCR and immunohistochemistry, BmorGR6 was detected in the midgut, central nervous system, and oral sensory organs, suggesting it acts as not only a GR but also a chemical sensor regulating gut movement, physiological conditions, and feeding behavior of larvae (Mang *et al*., 2016a). In addition, BmorGR6 and BmorGR9 were colocalized with cells in the brain, suggesting BmorGR6 may be involved in the regulation of feeding behaviors in *B. mori* larvae (Mang *et al*., 2016b). Membrane topology studies on BmorGR8 predicted an inverted topology relative to GPCR (Fig. 2) (Zhang *et al*., 2011). BmorGR8 functions independently in Sf9 cells and responds in a dose‐dependent manner to the polyalcohols *myo*‐inositol and *epi*‐inositol, important or essential nutrients for some Lepidoptera (Zhang *et al*., 2011). Eight *H. armigera* sugar *Gr* genes (*HarmGr4–8*, *10–12*) were identified and they have shown high, selective, and specific expressions in major gustatory tissues (Xu *et al*., 2017). Interestingly, all eight *H. armigera* sugar GRs are localized in a tandem array on the same scaffold of the genome, suggesting they may evolve from the same ancestral gene (Xu *et al*., 2017). HarmGR10 is specifically expressed in male adult testes while HarmGR11 is specifically expressed in female adult ovaries (Xu *et al*., 2017). Thus, HarmGR10 and HarmGR11 may play a role in nutrient sensation and regulate reproductive behaviors (Xu *et al*., 2017). *Drosophila* sugar GRs form functional heteromultimers *in vivo* (Jiao *et al*., 2008), indicating that coexpression of multiple GRs is essential for the detection of compounds such as sucrose, D‐glucose, and trehalose (Jiao *et al*., 2008). However, Lepidopteran sugar receptor seems to function independently in insect cell (Sf9), mammalian cell (HEK293T), or *Xenopus* oocyte system, suggesting they may detect sugars using a different mechanism.

### GR43a‐like receptors

GR43a is a conserved GR that exists in almost all insect species, and therefore may have a conserved function across insect orders. In *Drosophila*, GR43a showed narrowly tuned responses to fructose (Sato *et al*., 2011) and was detected in the brain (Miyamoto *et al*., 2012). *Drosophila* GR43a is crucial to sense hemolymph fructose and promote feeding in hungry flies but suppress feeding in satiated flies. Therefore, GR43a may function as internal nutrient sensors playing important roles in feeding behaviors (Miyamoto *et al*., 2012). BmorGR9, a GR43a ortholog in *B. mori*, was highly expressed in the gut (Sato *et al*., 2011). By immunohistochemistry, BmorGR9 was also detected the expression in cells of oral sensory organs including maxillary galea, maxillary palps, labrum, and labium as well as in putative neurosecretory cells of the central nervous system and brain, suggesting it is involved in promotion of feeding behaviors (Sato *et al*., 2011). *Xenopus* oocytes or HEK293T cells expressing BmorGR9 selectively responded to D‐fructose (Sato *et al*., 2011). Another GR43a ortholog from *B. mori*, BmorGR10 was detected expression in *B. mori* mouthparts such as maxillary galea, maxillary palp, and labrum (Kikuta *et al*., 2016). By using *Xenopus* oocyte expression system and mammalian cells coupled with calcium imaging techniques, BmorGR10 showed response to *myo*‐inositol, suggesting it may play an important role in the *myo*‐inositol recognition (Kikuta *et al*., 2016). HarmGR9, a GR43a homologue, was highly detected in the larval foregut of *H. armigera*, where food is stored before moving into the midgut for digestion (Xu *et al*., 2012). HarmGR9 was also detected in the gustatory sensory neuron of the contact chemosensilla on the distal part of the antenna, which was characterized sensilla chaetica responding to fructose (Jiang *et al*., 2015). After expressing HarmGR9 in Sf9 cells, calcium imaging analysis showed it responded to not only D‐fructose but also D‐galactose and D‐maltose (Xu *et al*., 2012). Interestingly, the electrophysiological responses of *Xenopus* oocytes expressing HarmGR9 showed a large response to D‐fructose only at a concentration of 0.050 mol/L. The D‐fructose‐induced current increased with fructose concentration from 0.005 mol/L to 0.300 mol/L (Jiang *et al*., 2015). Topology studies on HarmGR9 predicted an intracellular N‐terminus and an extracellular C‐terminus (Fig. 2), which is consistent with the study on *B. mori* GRs (Zhang *et al*., 2011). Swallowtail butterflies (*Papilio xuthus*) utilize a limited number of plants to lay eggs. PxutGR1, a GR43a‐like receptor from female tarsi of *P. xuthus*, was expressed in Sf9 cells and showed response specifically to synephrine, a plant alkaloid. *P. xuthus* sensitivity of tarsal taste sensilla to synephrine and the oviposition behavior in response to synephrine were strongly decreased after RNAi knock‐down of PxutGR1, suggesting PxutGR1 is a key factor in host specialization in *P. xuthus* (Ozaki *et al*., 2011).

### Bitter receptors

Bitter is a taste modality generally associated with toxic substances evoking aversive behaviors in insects. Relatively few studies have investigated insect bitter taste receptors except *Drosophila* (Moon *et al*., 2006; Lee *et al*., 2009; Weiss *et al*., 2011). The Lepidopteran bitter GRs were characterized into three “types” based on gene structure and length (Fig. 2). Type 1 exhibits structural features, which are conserved across Lepidoptera species while Type 2 and Type 3 are intronless GRs that are less commonly found in the available Lepidopteran genomes. Type 2 refers to those genes that are relatively long (>400 AA) and Type 3 as those that are relatively short (<360 AA). Most of the bitter receptors in *H. armigera* fall into the Type 3 category (Xu *et al*., 2016), which are often arranged in gene clusters in the genome, suggesting they arise from a few ancestral genes that have undergone successive duplications (Xu *et al*., 2016).

Insect GRs were predicted to have seven transmembrane domains (TMDs), an intracellular N‐terminus, and an extracellular C‐terminus (Wanner & Robertson, 2008; Zhang *et al*., 2011). Two Type 2 GRs, BmorGR53 and HarmGR65, showed the consistent topologies in Sf9 cells (Zhang *et al*., 2011; Xu *et al*., 2016) as expected (Fig. 2). However, two different topologies were detected from two Type 3 bitter GRs, HarmGR35 and HarmGR50. HarmGR35 has two intracellular termini while HarmGR50 has two extracellular termini (Fig. 2). Type 3 GRs are also predicted to have fewer TMDs, making them with multiple topologies (Xu *et al*., 2016). *Drosophila* GRs have been shown to form functional heteromultimers *in vivo* (Jones *et al*., 2007; Lee *et al*., 2009; Shim *et al*., 2015). Type 3 bitter receptors were able to function alone in Sf9 cells; however, it is possible that multiple GRs are required for function *in vivo* (Xu *et al*., 2016).

By using calcium imaging in mammalian cells, two Type 1 bitter receptors (BmorGR16 and BmorGR18) and one Type 2 bitter receptor (BmorGR53) from *B. mori* were confirmed widely responded to structurally different feeding deterrents such as coumarin and caffeine, suggesting their roles in the host plant recognition (Kasubuchi *et al*., 2018). All these three GRs are expressed in the labrum, maxillary palp, and maxillary galea at different levels (Kasubuchi *et al*., 2018). Three Type 3 bitter receptors from *H. armigera*, HarmGR35, HarmGR50, and HarmGR195, showed responses to the crude extract of cotton leaves. Further, HarmGR195, which is specifically expressed in the adult tarsi, responded to proline in a dose‐dependent manner (Xu *et al*., 2016). Plant nectars may contain up to 2 mmol/L proline (Carter *et al*., 2006), and this high level of proline is thought to be an attractant, as several species of insects prefer high‐proline nectars (Carter *et al*., 2006). Given that it is highly expressed on the tarsi, HarmGR195 may have a role in regulating the insects’ feeding or oviposition (Xu *et al*., 2016).

One *B. mori* Type 1 bitter GR, BmorGR66, was genetically mutated by using CRIRPR/Cas9 system *in vivo*. The mutated silkworm larvae lost their specificity for mulberry and started to feed on fruits and grain seeds, indicating a single bitter GR is a major factor affecting insect feeding behaviors (Zhang *et al*., 2019).

## Perspectives

Lepidopteran GRs are the potential molecular targets that shed light on the development of new insect control pathways. GRs have been reported to play a critical role in regulating behaviors and developmental signaling pathways. Thus, blocking or disruption of these receptors may have marked behavioral, developmental phenotypic deformative effects, which may lead to enhanced mortality of insects. For example, a class of odorants present in food was identified to inhibit CO_2_‐sensitive neurons in *Drosophila* (Turner & Ray, 2009). Furthermore, the advanced gene‐knockout and knock‐down techniques such as CRISPR/Cas and RNAi have been successfully applied to insect GRs to characterize their functions. By interfering with GRs in the insect gustatory system, we may find new strategies to reduce the capability of insect pests to damage and transmit diseases to human, animal, and crops. For example, after injection of *NlGr11* double‐stranded RNA into brown planthopper (BPH), *Nilaparvata lugens*, the number of eggs laid by BPH decreased significantly, indicating that a GR modulates the fecundity of insects and that the receptor could be a potential target for pest control (Chen *et al*., 2019).

Furthermore, insect GRs can be used to screen new attractants or repellents. For example, DEET is the most widely used insect repellent worldwide. DEET has been shown to suppress feeding behavior, which was mediated by GRs in *Drosophila* (Lee *et al*., 2010). Therefore, the interest GRs can be used to “fish” new attractant or repellent candidates. Especially the recently published insect odorant receptor coreceptor (Orco) 3D structure provides new insights into the enormous diversity of insect chemosensory receptors and their specificity and selectivity to the ligands (Butterwick *et al*., 2018; Luo & Carlson, 2018). It is expected that more and more insect GR structures will be studied to explore GR‐ligand interactions at molecular level, which will provide insights into the mechanisms and functions of GRs to optimize current attractants or repellents.

Last but not the least, biosensors based on insect GRs will be another future research direction for detecting chemical compounds. Lepidopteran GRs are ideal models to study the mechanism of ligand–receptor interactions. Unlike insect ORs, which require the Orco to form heteromeric complexes to function, Lepidopteran GRs (except CO_2_ receptors) have been shown to function independently in insect cell, mammalian cell or *Xenopus* oocytes. Therefore, they have the potential to develop next generation of biosensors, which can be used broadly in agriculture, horticulture, health, and security.

## Disclosure

The authors declare no conflict of interest.
